# Nanoparticle albumin-bound paclitaxel and PD-1 inhibitor (sintilimab) combination therapy for soft tissue sarcoma: a retrospective study

**DOI:** 10.1186/s12885-022-09176-1

**Published:** 2022-01-12

**Authors:** Zhichao Tian, Shuping Dong, Yang Yang, Shilei Gao, Yonghao Yang, Jinpo Yang, Peng Zhang, Xin Wang, Weitao Yao

**Affiliations:** 1 Department of Bone and Soft Tissue, The Affiliated Cancer Hospital of Zhengzhou University and Henan Cancer Hospital, Dongming Road, Zhengzhou, 450008 Henan Province China; 2 Huanghe Science and Technology College, Zhengzhou, 450063 Henan Province China; 3 Department of Immunotherapy, The Affiliated Cancer Hospital of Zhengzhou University and Henan Cancer Hospital, Zhengzhou, 450008 Henan Province China; 4 Department of Medical Oncology, The Affiliated Cancer Hospital of Zhengzhou University and Henan Cancer Hospital, Zhengzhou, 450008 Henan Province China

**Keywords:** Nanoparticle albumin-bound paclitaxel, PD-1 inhibitor, Programmed cell death protein 1, Sintilimab, Soft tissue sarcoma, Angiosarcoma

## Abstract

**Background:**

There is increasing evidence that combination therapy with nanoparticle albumin-bound paclitaxel (nab-paclitaxel) and programmed cell death protein 1 (PD-1) inhibitor is safe and efficacious in treating many types of malignant tumors. However, clinical data demonstrating the effect of this treatment combination for patients with metastatic soft tissue sarcoma (STS) are currently limited.

**Methods:**

The clinical data of patients with metastatic STS who received nab-paclitaxel plus PD-1 inhibitor (sintilimab) therapy between January 2019 and February 2021 were retrospectively analyzed. The effectiveness and safety of the combined treatment were evaluated in terms of the median progression-free survival (PFS), estimated using the Kaplan–Meier method. The univariate Cox proportional hazards model was used to analyze the relationship between clinicopathological parameters and PFS. All statistical analyses were two-sided; *P* < 0.05 was considered statistically significant.

**Results:**

A total of 28 patients treated with nab-paclitaxel plus sintilimab were enrolled in this study. The objective response rate was 25%, the disease control rate was 50%, and the median PFS was 2.25 months (95% CI = 1.8–3.0 months). The most common grade 1 or 2 adverse events (AEs) were alopecia (89.3%; 25/28), leukopenia (25.0%; 7/28), fatigue (21.4%; 6/28), anemia (21.4%; 6/28), and nausea (21.4%; 6/28). The most common grade 3 AEs were neutropenia (10.7%; 3/28) and peripheral neuropathy (10.7%; 3/28). No grade 4 AEs were observed. Among the present study cohort, patients with angiosarcoma (*n* = 5) had significantly longer PFS (*P* = 0.012) than patients with other pathological subtypes, including undifferentiated pleomorphic sarcoma (*n* = 7), epithelioid sarcoma (*n* = 5), fibrosarcoma (*n* = 4), synovial sarcoma (*n* = 3), leiomyosarcoma (*n* = 2), pleomorphic liposarcoma (*n* = 1), and rhabdomyosarcoma (*n* = 1); those who experienced three or more AEs had significantly longer median PFS than those who experienced less than three AEs (*P* = 0.018).

**Conclusion:**

Nab-paclitaxel plus PD-1 inhibitor is a promising treatment regimen for advanced STS. Randomized controlled clinical trials are required to further demonstrate its efficacy and optimal application scenario.

## Background

Soft tissue sarcomas (STSs) are malignant tumors originating from the mesenchymal tissue. This type of tumor occurs throughout the body and is typically characterized by an asymptomatic mass. Some STSs that grow too fast can cause pain by pressing on the surrounding tissue [1]. The preferred treatment for early and middle-stage STS is complete resection [2]. Approximately 50% of STSs metastasize primarily to the lungs via blood circulation despite surgery [3]. Although STS incidence is low (approximately 4 per 100,000), there are over 70 subtypes [1, 4]. Despite each subtype of STS having different sensitivity to radiotherapy or chemotherapy, the first- and second-line chemotherapy regimen for advanced STS is doxorubicin and docetaxel plus gemcitabine, respectively [2, 3]. However, the response rate for the aforementioned regimens is < 20%, resulting in a median overall survival of approximately 12 months for patients with advanced STS [5–7]. Therefore, more effective therapies are urgently needed.

Nanoparticle albumin-bound paclitaxel (nab-paclitaxel) is a new type of taxane antineoplastic drug [8]. Compared with the other two major taxanes, paclitaxel and docetaxel, nab-paclitaxel is more water-soluble and bioavailable, and less toxic, thus improving its anti-tumor efficacy [8–10]. Nab-paclitaxel has been increasingly used to treat various types of malignant tumors [11–13]. Several recent reports suggest that nab-paclitaxel is effective in treating sarcomas and proved more effective than docetaxel, a second-line treatment for STS, in some subtypes [14–16].

As a new type of anti-tumor therapy, anti-programmed cell death protein 1 (PD-1) inhibitors have been widely used in STS treatment and research [17]. Although recent evidence shows that the response rate of PD-1 inhibitor monotherapy is low in patients with STS [17, 18], there have been encouraging reports of efficacy in some sarcoma subtypes [19]. The combination of PD-1 inhibitors with cytotoxic chemotherapy agents is a promising way to improve the efficacy of PD-1 inhibitors in the treatment of patients with malignant tumors, including STS [18, 20–22].

Combination therapy with nab-paclitaxel and PD-1 inhibitors has proven effective in treating patients with advanced lung cancer and melanoma [23–25]. However, the efficacy and safety of nab-paclitaxel combined with a PD-1 inhibitor for patients with STS have not been reported. We have extensive experience in the treatment of patients with advanced STS using nab-paclitaxel or PD-1 inhibitors [26, 27]. Some patients were treated with nab-paclitaxel plus a PD-1 inhibitor. In this study, we retrospectively collected and analyzed the clinical data of patients with advanced STS who were treated with nab-paclitaxel plus a PD-1 inhibitor to provide reference data for the diagnosis, treatment, and clinical trial design of advanced STS.

## Methods

### Patient enrolment and eligibility criteria

This was a retrospective study of patients with STS treated at the Affiliated Cancer Hospital of Zhengzhou University (Zhengzhou, China). All the patients received nab-paclitaxel plus a PD-1 inhibitor between January 2019 and February 2021. The patient eligibility criteria included: 1) histologically proven STS, 2) treatment with nab-paclitaxel plus PD-1 inhibitor, 3) locally unresectable or multiple metastases, 4) measurable lesions according to the response evaluation criteria in solid tumors (RECIST; version 1.1) [28], and 5) complete clinical data and statistical analysis. This analysis was descriptive, and the follow-up was extended to June 30, 2021.

### Treatment protocol

The patients received 300 mg/m^2^ of nab-paclitaxel (Hengrui Pharmaceutical, Lianyungang, China) and 200 mg of PD-1 inhibitor (sintilimab; Innovent Biologics, Suzhou, China) via a 30-min intravenous infusion on day 1. The treatment was repeated every 3 weeks until progressive disease (PD) occurrence or unacceptable adverse events (AEs). If grade 3 or 4 AEs occurred, treatment was delayed for a maximum of 14 days until recovery.

### Evaluation of AEs and tumor responses

We firstly reviewed the baseline the demographics and characteristics of STS patients enrolled in this study. According to the RECIST (version 1.1), the tumor responses evaluation was performed every 6 weeks, or immediately when there is a clear signal of PD was observed. Tumor responses were categorized as PD, stable disease (SD), partial response (PR) or complete response (CR). The objective response rate (ORR) was defined as the sum of CR and PR rates. Disease control rate (DCR) was defined as the sum of the ORR and SD. PFS was defined the time from the date of the first nab-paclitaxel plus PD-1 inhibitor treatment to the date of PD was observed. The National Cancer Institute Common Terminology Criteria for Adverse Events (version 4.0) was used to assess the AEs. Finally, the relationship between clinical parameters and PFS was assessed.

### Statistical analysis

Quantitative variables are presented as numerical values (percentages) and medians (ranges). Statistical analyses were performed using SPSS 21.0 (SPSS Inc., Chicago, IL, USA). The corresponding figures were drawn using GraphPad Prism 5.0 (GraphPad Software Inc., San Diego, CA, USA). PFS was estimated using the Kaplan–Meier method. Univariate Cox proportional hazards model was used to analyze the relationship between the clinicopathological parameters and PFS. All statistical analyses were two-sided, and a *P* value of < 0.05 was considered statistically significant.

## Results

### Patient characteristics

From January 2019 to February 2021, 28 patients (average age ± standard deviation = 42.54 ± 14.31 years) with advanced STS, treated with nab-paclitaxel plus PD-1 inhibitor, were identified. The cohort included 13 men and 15 women (Table 1); all had an Eastern Cooperative Oncology Group performance status of 0 or 1 and stage IV disease. The histological subtypes included undifferentiated pleomorphic sarcoma (*n* = 7), angiosarcoma (*n* = 5), epithelioid sarcoma (*n* = 5), fibrosarcoma (*n* = 4), synovial sarcoma (*n* = 3), leiomyosarcoma (*n* = 2), pleomorphic liposarcoma (*n* = 1), and rhabdomyosarcoma (*n* = 1). The primary tumor site was distributed throughout the body, but mainly in the extremities. Lung metastasis occurred first in most patients, and all had previously received 1–3 lines of chemotherapy (Table 1).

**Table 1:** Patient demographics and characteristics

Patient No.	ECOG PS	Histological subtype	Stage	Primary site	Metastatic site	Previous lines of chemotherapy	Response	PFS (Months)
1	1	UPS	IV	Head	Lung and bone	1	PR	5
2	1	UPS	IV	Extremities	Lung and lymph nodes	1	PR	3.5
3	0	UPS	IV	Extremities	Lung and bone	2	SD	3
4	0	UPS	IV	Neck	Bone	2	SD	2.5
5	0	UPS	IV	Extremities	Lung	2	PD	2
6	0	UPS	IV	Extremities	Bone	2	PD	1.8
7	1	UPS	IV	Extremities	Lung	1	PD	1
8	1	Angiosarcoma	IV	Head	Liver	2	CR	15
9	0	Angiosarcoma	IV	Trunk	Bone	2	PR	7.8
10	1	Angiosarcoma	IV	Extremities	Lung	2	PR	4
11	0	Angiosarcoma	IV	Extremities	Lung	2	SD	4.5
12	0	Angiosarcoma	IV	Trunk	Lung	2	PD	2
13	1	Epithelioid sarcoma	IV	Extremities	Lung	2	PR	2.8
14	1	Epithelioid sarcoma	IV	Trunk	Lung and lymph nodes	2	SD	3
15	1	Epithelioid sarcoma	IV	Trunk	Lung	2	SD	2.8
16	0	Epithelioid sarcoma	IV	Extremities	Lung	1	PD	1.8
17	1	Epithelioid sarcoma	IV	Extremities	Lung and bone	2	PD	1.3
18	1	Fibrosarcoma	IV	Trunk	Lung	2	PR	4.3
19	1	Fibrosarcoma	IV	Extremities	Lung	2	SD	2.5
20	0	Fibrosarcoma	IV	Extremities	Lung	1	PD	1.5
21	0	Fibrosarcoma	IV	Extremities	Liver	3	PD	1.3
22	0	Synovial sarcoma	IV	Neck	Lung	2	PD	1.8
23	0	Synovial sarcoma	IV	Extremities	Lung	2	PD	1.5
24	1	Synovial sarcoma	IV	Trunk	Lung	3	PD	1.5
25	0	Leiomyosarcoma	IV	Extremities	Lung	2	SD	2.8
26	1	Leiomyosarcoma	IV	Trunk	Liver	1	PD	1.3
27	0	Pleomorphic liposarcoma	IV	Extremities	Lung	2	PD	1.8
28	1	Rhabdomyosarcoma	IV	Head	Lung	1	PD	1.5

The clinical and pathologic stages are expressed according to the American Joint Committee on Cancer (8th Edition) TNM staging. Tumor responses were evaluated according to the Response Evaluation Criteria in Solid Tumors (version 1.1), and were categorized as *CR* Complete response, *PR* Partial response, *SD* Stable disease, or *PD* Progressive disease. *PFS* Progression-free survival was calculated from the date of the first nab-paclitaxel plus PD-1 inhibitor treatment until the date of documented progression

*Abbreviations*: *ECOG PS* Eastern Cooperative Oncology Group performance status, *UPS* Undifferentiated pleomorphic sarcoma

### Effectiveness of therapy

**Fig. 1:**
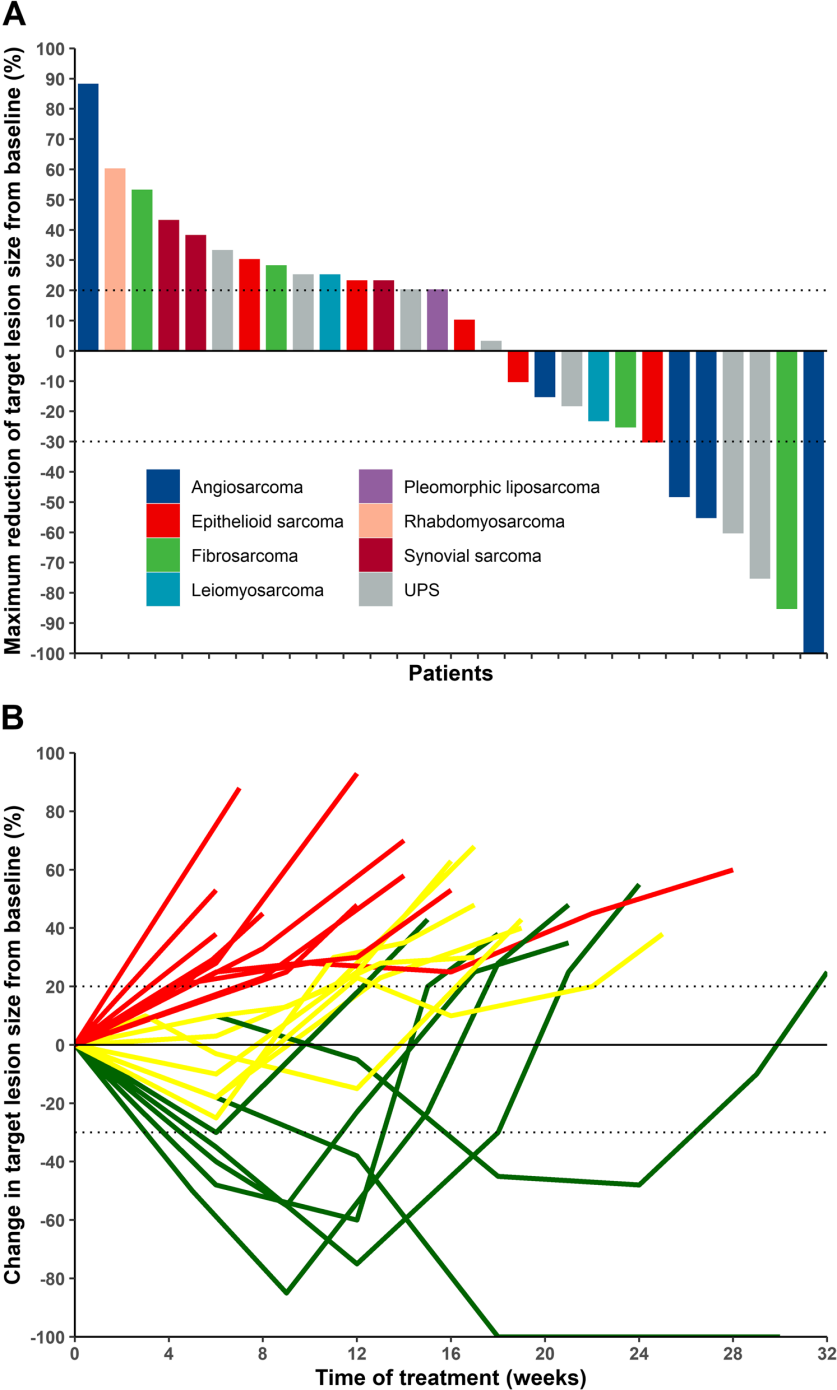
Target lesion changes in patients with soft tissue sarcoma treated with nanoparticle albumin-bound paclitaxel plus a programmed cell death protein 1 inhibitor. **A** Waterfall plot shows the maximum reduction of target lesion size from baseline evaluated according to the Response Evaluation Criteria for Solid Tumors (RECIST, version 1.1). The horizontal axis represents different patients, and the vertical axis represents the percentage of change in the target lesions. One patient with angiosarcoma had a complete response (100% decrease in target lesion size), six patients had a partial response (30% and more decrease in target lesion size), seven patients had stable disease (< 20% increase and < 30% decrease in target lesion size), and 14 patients had progressive disease (20% and more increase in target lesion size). **B** Line plot shows the duration of response of the target lesions from baseline evaluated according to RECIST (version 1.1). Each line represents the change in the size of a patient’s target lesion over the time of treatment. Patients with a complete or partial response are indicated in green, those with progressive disease in red, and those with stable disease in yellow

Of the 28 patients with advanced STS, one patient with angiosarcoma had a CR, six had a PR, and seven had SD (Tables 1 and 2; Fig. 1). The ORR, DCR, median-PFS, and 4-month PFS rate were 25, 50%, 2.25 months (95% CI; 1.8–3.0 months), and 17.9% (Table 3; Fig. 1), respectively.

**Table 2:** Responses of various histological subtypes to treatment

Histological subtypes	Number of patients			
	CR	PR	SD	PD
UPS (*n* = 7)	0	2	2	3
Angiosarcoma (*n* = 5)	1	2	2	0
Epithelioid sarcoma (*n* = 5)	0	1	2	2
Fibrosarcoma (*n* = 4)	0	1	1	2
Synovial sarcoma (*n* = 3)	0	0	0	3
Leiomyosarcoma (*n* = 2)	0	0	1	1
Pleomorphic liposarcoma (*n* = 1)	0	0	0	1
Rhabdomyosarcoma (*n* = 1)	0	0	0	1
Total	1	6	8	13

Tumor responses were evaluated according to the Response Evaluation Criteria in Solid Tumors (version 1.1), and were categorized as *CR* Complete response, *PR* Partial response, *SD* Stable disease, or *PD* Progressive disease

*Abbreviations*: *UPS* Undifferentiated pleomorphic sarcoma

**Table 3:** Clinical effectiveness

Characteristics	Data
ORR (%)	25.00 (95%CI: 10.7-44.9)
DCR (%)	50.00 (95%CI: 30.6-69.4)
M-PFS (months)	2.25(95%CI: 1.80-3.00)
4 months PFS rate (%)	17.9 (95%CI: 0.081-0.395)
6 months PFS rate (%)	7.1 (95%CI: 0.019-0.272)

Data are presented as percentages or means. Tumor responses were evaluated according to the Response Evaluation Criteria in Solid Tumors (version 1.1), and were categorized as *CR* Complete response, *PR* Partial response, *SD* Stable disease, or progressive disease. The *ORR* Objective response rate was defined as the sum of CR and PR rates. *DCR* Disease control rate was defined as the sum of the ORR and SD. *PFS* Progression-free survival was calculated from the date of the first nab-paclitaxel plus PD-1 inhibitor treatment until the date of documented progression

### Toxicity and safety

In general, nab-paclitaxel plus PD-1 inhibitor therapy was relatively well tolerated (Table 4). The most common grade 1 or 2 AEs were alopecia (89.3%; 25/28), leukopenia (25.0%; 7/28), fatigue (21.4%; 6/28), anemia (21.4%; 6/28), and nausea (21.4%; 6/28). The most common grade 3 AEs were neutropenia (10.7%; 3/28) and peripheral neuropathy (10.7%; 3/28). No grade 4 AEs were observed. None of the patients received a reduced dose of nab-paclitaxel or PD-1 inhibitor due to AEs, and no treatment-related deaths occurred.

**Table 4:** Adverse events

Adverse events	Grade 1-2	Grade 3-4
Alopecia	89.3% (25/28)	
Leucopenia	25.0% (7/28)	10.7% (3/28)
Fatigue	21.4% (6/28)	3.6% (1/28)
Anemia	21.4% (6/28)	
Nausea	21.4% (6/28)	3.6% (1/28)
Peripheral neuropathy	17.9% (5/28)	10.7% (3/28)
Transaminase increase	17.9% (5/28)	7.1% (2/28)
Anorexia	14.3% (4/28)	3.6% (1/28)
Diarrhea	14.3% (4/28)	3.6% (1/28)
Thrombocytopenia	14.3% (4/28)	
Hypothyroidism	14.3% (4/28)	3.6% (1/28)
Pneumonitis	10.7% (3/28)	3.6% (1/28)
Fever	10.7% (3/28)	
Abdominal pain	7.1% (2/28)	
Rash	3.6% (1/28)	

Data are presented as percentages (number events/total). Adverse events were assessed using the National Cancer Institute Common Terminology Criteria for Adverse Events (version 4.0)

### Univariate Cox regression analysis

**Fig. 2:**
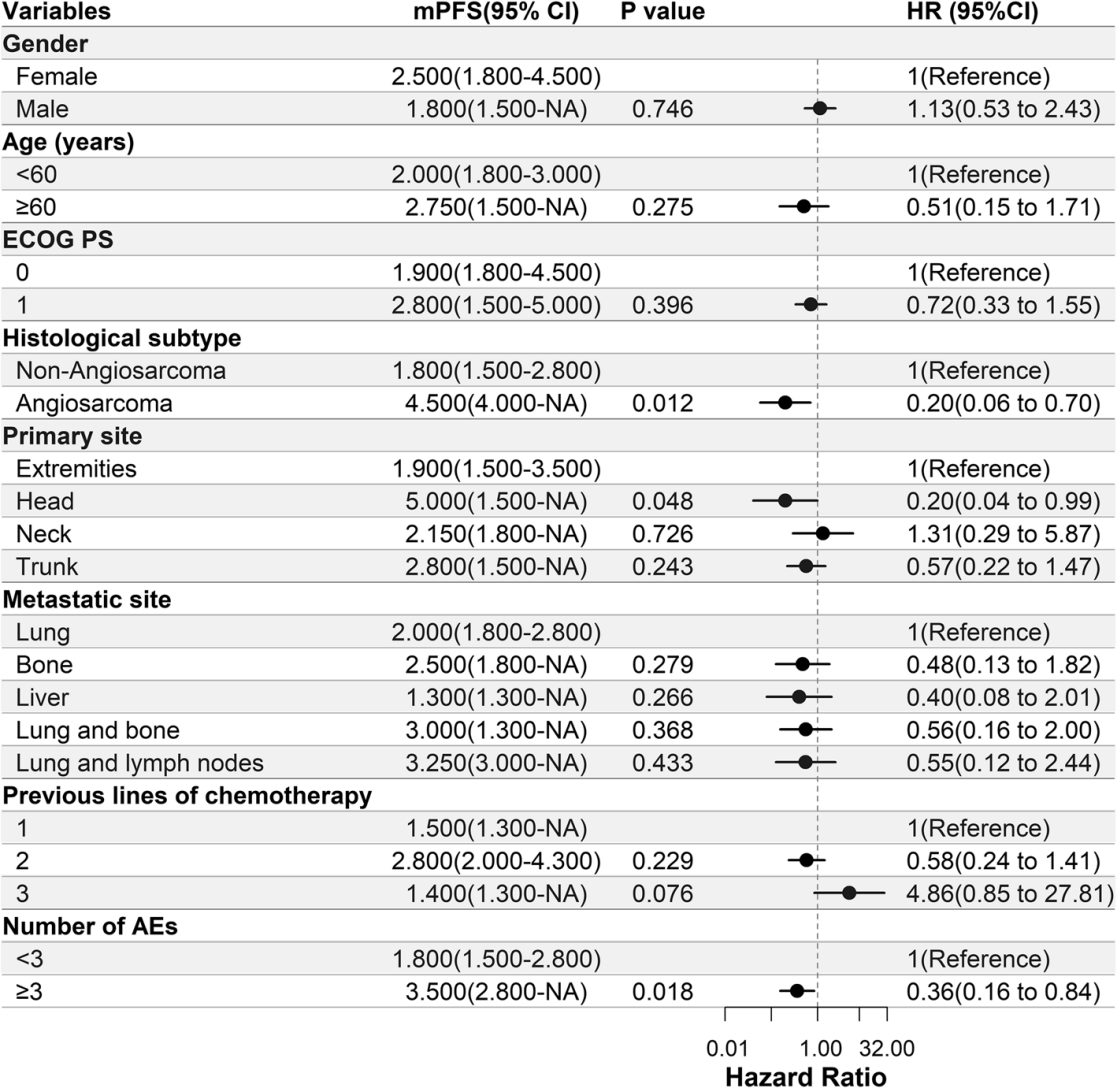
Univariate Cox regression analysis of the relationship between clinicopathological parameters and progression-free survival (PFS). When the hazard ratio (HR) (95% CI) of a factor is completely on the right side of the dotted line, it means that it is a risk factor; When it is completely to the left of the dotted line, it means that it is a protective factor; When the dotted line is included, it cannot be judged whether it is a risk factor or a protective factor. Among the patients in this study, those with angiosarcoma had a significantly longer PFS compared to those with other pathological subtypes (HR = 0.20, 95%CI 0.06 - 0.70, *P* = 0.012); those with the primary tumor site in the head region had a significantly longer PFS than those with the primary tumor at other sites (HR = 0.20, 95%CI 0.04 - 0.99, *P* = 0.048); those who experienced three or more adverse events (AEs) had significantly longer PFS than those who experienced less than three AEs (HR = 0.36, 95%CI 0.16 - 0.84, *P* = 0.018). ECOG PS, eastern cooperative oncology group performance status; mPFS, median PFS

Univariate Cox regression analysis was performed to determine the relationship between the median PFS and clinical characteristics of the patients in this study (Fig. 2). Among our patient cohort, those with angiosarcoma had a significantly longer PFS than those with other pathological subtypes (HR = 0.20, 95%CI 0.06 - 0.70, *P* = 0.012); those with the primary tumor site in the head region had a significantly longer PFS than those with the primary tumor at other sites (HR = 0.20, 95%CI 0.04 - 0.99, *P* = 0.048); those who experienced three or more AEs had a significantly longer PFS than those who experienced less than three AEs (HR = 0.36, 95%CI 0.16 - 0.84, *P* = 0.018).

## Discussion

In this study, we retrospectively reviewed and analyzed the clinical data of 28 patients with advanced STS who received nab-paclitaxel plus PD-1 inhibitor (sintilimab) treatment. The ORR, DCR, and median PFS were 25, 50%, and 2.25 months, respectively. In general, nab-paclitaxel plus PD-1 inhibitor therapy was relatively well tolerated. Univariate Cox regression analysis showed that patients with angiosarcoma or who experienced three or more AEs had significantly longer PFS.

To the best of our knowledge, this study is the first to report the effectiveness of nab-paclitaxel plus PD-1 inhibitors for the treatment of advanced STS. Since the efficacy of PD-1 inhibitor monotherapy in STSs is extremely low, the use of various methods to reprogram the tumor microenvironment from immune-“cold” to immune-“hot” to increase the sensitivity of PD-1 inhibitors in STS is a promising approach [18, 20]. Potential mechanisms of the combination therapy of PD-1 inhibitors with cytotoxic chemotherapy agents include immunogenic tumor cell death, anti-angiogenesis, selective depletion of myeloid immunosuppressive cells, reduction of tumor-induced immune suppression, and the sensitization of tumor cells to the immune response [20, 29].

Chemotherapy combined with a PD-1 inhibitor has been shown to have a beneficial effect against various other malignancies [20, 25, 29]. Recent clinical trials have demonstrated the efficacy and safety of the combination of the PD-1 inhibitor, pembrolizumab, and the chemotherapy drug, doxorubicin, for treating patients with advanced STS [22, 30]. These results suggest that it is feasible to improve the sensitivity of PD-1 inhibitors by combining them with chemotherapeutic agents. Nab-paclitaxel is a well-known inducer of immunogenic cell death and has been shown to improve the efficacy of PD-1 inhibitors via the regulation of various immune functions [25, 31]. The synergistic effect of nab-paclitaxel combined with a PD-1 inhibitor has been demonstrated [23–25, 32]. Therefore, the aforementioned combination regimen was selected for the treatment of our patients.

The results of this retrospective study showed that the combination regimen was significantly more effective against advanced STS than PD-1 inhibitor monotherapy [19]. Furthermore, our approach was as effective as the use of paclitaxel plus gemcitabine for STS treatment [26, 33], indicating that the combination of nab-paclitaxel and a PD-1 inhibitor improves the sensitivity of both the PD-1 inhibitor and nab-paclitaxel toward STS and provides a reference for the further study of this combination regimen. Although the combination regimen in this study was as effective as the administration of paclitaxel plus gemcitabine in STS treatment, the use of nab-paclitaxel plus PD-1 inhibitor has its unique benefits. For instance, the infusion conditions required by nab-paclitaxel or PD-1 inhibitors are simple and convenient, which are important considerations for patient satisfaction. Another important finding was that the combination therapy appeared to have greater efficacy against certain subtypes of sarcomas, particularly angiosarcoma. Therefore, nab-paclitaxel plus PD-1 inhibitor is a strong complement to existing therapies for STSs. However, it should be noted that the median PFS was relatively short for the patients in this study. This may be attributed to the small sample size or a potential problem with the treatment regimen.

In terms of safety, the incidence of AEs was low among patients in this study; grade 3–4 AEs were rare. This suggests that the nab-paclitaxel plus PD-1 inhibitor combination has a favorable safety profile, which is in accordance with the findings of previous studies [24, 32]; the safety profile of this combination regimen is significantly better than that of paclitaxel plus gemcitabine for STS therapy [26, 33]. This can greatly improve the satisfaction and quality of life of patients undergoing treatment. In addition, univariate analysis showed that patients who experienced a higher number of AEs had longer PFS. This is similar to the results of another study on patients with refractory melanoma, in which significantly longer median PFS (*P* < 0.05) was reported for patients who experienced ≥3 AEs or immune-related AEs during combination therapy with nab-paclitaxel and anti-PD-1 inhibitor [25]. In other words, patients who experienced fewer AEs had poorer outcomes. Therefore, it may be considered to increase the dose of the drug in patients with a low incidence of AEs, thus improving the therapeutic effect.

Our study had some limitations. This includes but is not limited to the relatively low number of patients and short follow-up time, as well as the lack of monitoring patients’ immune status during the treatment period. Furthermore, this study also identified some problems that require investigation. First, it is still unknown which drug plays a major role in this combination. Moreover, the potential synergistic mechanisms between these two drugs should be elucidated. The optimal dosage regimen for nab-paclitaxel is also not yet clear. Finally, given that the effect of this combination therapy varied among subtypes of STSs, further clinical studies with larger sample sizes should be conducted to determine which patients would benefit most from this treatment protocol.

## Conclusions

The results of this study demonstrated that a combination of nab-paclitaxel plus PD-1 inhibitor is a promising treatment regimen for advanced STS, and it is worth conducting randomized controlled clinical trials to further demonstrate its efficacy and determine its optimal application scenario.

## Data Availability

The datasets used and/or analyzed in the current study are available from the corresponding author on reasonable request.

## Abbreviations

AE: Adverse events
CR: Complete response
DCR: Disease control rate
nab-paclitaxel: Nanoparticle albumin-bound paclitaxel
ORR: Objective response rate
PD: Progressive disease
PD-1: Programmed cell death protein 1
PFS: Progression-free survival
PR: Partial response
RECIST: Response evaluation criteria in solid tumors
SD: Stable disease
STS: Soft tissue sarcoma
